# The power of webinars: expanding diverse female representation and role models in surgery

**DOI:** 10.1186/s12909-026-09326-0

**Published:** 2026-05-01

**Authors:** Maria Prayle, Isobel Seddon, Helen Wallace

**Affiliations:** 1 Mersey West Lancashire Hospital Trust, Liverpool, UK; 2 NHS Lothian, Edinburgh, UK; 3 Department of Women’s and Children’s Health, University of Liverpool, Liverpool, UK

**Keywords:** Women in Surgery, Widening Participation, Diversity, Role Models, Equality, Surgery, Representation, Medical Education

## Abstract

**Background:**

Women, particularly those from widening participation (WP) backgrounds, remain underrepresented in surgical specialties. The educational pipeline is marked by limited exposure to relatable role models, which may discourage these individuals from pursuing surgical careers. Digital learning platforms offer a novel approach to address this gap.

**Methods:**

In collaboration with the Widening Participation Medics Network (WPMN), a UK-based charity supporting underrepresented medical students and doctors, we delivered a series of five free webinars. Each featured female surgeons—many from WP backgrounds—discussing surgical careers and personal barriers faced. Attendees ranged from pre-medical students to senior clinicians. Post-event surveys (*n* = 275, 75% response rate) collected both numerical and free-text responses to evaluate impact on understanding, motivation, and representation.

**Results:**

The series reached 369 live attendees globally, with 60% identifying as WP and 27% reporting multiple WP characteristics. 73% of attendees agreed that there are many barriers preventing women from widening participation backgrounds entering into a surgical career and thriving as a surgeon. Exposure to WP-focused content of the webinars significantly improved insight into the challenges women face in surgery (Wilcoxon signed-rank test, *p* < 0.001, *r* = 0.74). A high proportion (84%) reported increased motivation to pursue surgery after attending the webinars. Presence of female speakers, surgical theme and WP narratives were the most cited reasons for attendance. Many (33%) had never previously attended an educational event featuring WP discussion.

**Conclusion:**

Webinars centered on lived experiences and diversity are effective in providing impactful surgical education and inspiration, especially for underrepresented students and doctors. These findings support the integration of inclusive, role model-led virtual teaching in medical education. Further longitudinal studies are needed to evaluate career progression and retention impacts.

**Supplementary Information:**

The online version contains supplementary material available at 10.1186/s12909-026-09326-0.

## Background

Current statistics show steady increases in female surgical trainees, however all surgical specialties remain highly male dominated with less than 30% of consultants being female [1]. This landscape of gender inequality is not exclusive to the UK [2]. Furthermore, intersectionality plays a role in preventing women from minority backgrounds reaching consultancy or holding leadership roles [3, 4].

There is now a growing body of evidence that shows that being operated on by a female surgeon or being cared for by a surgical team with more women reduces post-operative complications, duration of hospital stay, readmission to hospital and mortality [5, 6]. Akin to other industries, having a diverse team with more women improves communication, performance and team satisfaction [7, 8].

Since the Kennedy report in 2021 [9], there has been a shift in focus toward increasing diversity and inclusivity by The Royal College of Surgeons and other surgical organisations. A growth of women in surgery initiatives has been observed such as the continued work and expansion of The Royal College of Surgeons of England’s Women in Surgery (WinS) Network [10] and medical school specific WinS programmes and societies. There is limited evidence to suggest what has been successful thus far in encouraging women from diverse backgrounds to become surgeons. An avenue which may pose success is increasing accessibility to female surgeons who may act as role models, by providing a platform through the use of webinars.

Widening participation (WP) is a term used predominantly in the UK context, adopted by government bodies and medical schools to describe initiatives aimed at increasing access to and success within medical careers for those from underrepresented socioeconomic groups [11]. They include individuals from lower socioeconomic backgrounds and those who attended schools with historically low progression rates into higher education. Diversity is also a key element of WP in higher education, including (but not limited to) those from ethnic minority communities, all genders, sexual orientation, age and disability. This study worked with Widening Participation Medics Network (WPMN), a rapidly growing charity founded in 2020 by and for widening participation (WP) students, medical students and doctors [12]. Its mission is to break down barriers at every stage of the medical journey through advocacy, resource-sharing, and mentorship. In light of the Kennedy report and the current surrounding concerns about diversity in surgery, this study (driven by WPMN volunteers) set out to evaluate by the use of a survey, if the creation and utilisation of free online webinars consisting of underrepresented WP women in surgery, challenges current stereotypes and inspires the next generation of women in surgery. The main aims of the study were to develop a webinar series to provide inspiring educational insights from successful female surgeons within various surgical specialties; to assess the current perceptions of women in surgery and the barriers that may exist; to determine the impact of webinars on inspiration, insight and increasing motivation surrounding surgery as a viable career, and to evaluate how the WP focussed content was received.

## Method

### Social context

The WP framework was selected for this study, given its direct alignment with WPMN’s charitable mission and its established recognition within UK medical education policy as a means of describing and addressing structural inequities in university admissions and medical workforce diversity. Within this study, WP backgrounds encompass a broad range of underrepresented groups, reflecting the evolving and inclusive application of this term in modern medical education. This includes individuals from lower socioeconomic groups, those who attended schools with low progression rates into higher education, first generation to attend higher education, those from ethnic minority backgrounds, carers, care leavers, those who identify as LGBTQ+, and those living with a disability.

### Webinar background and structure

In 2022, WPMN developed an educational webinar series designed for WP Women in Surgery. This initiative featured a series of five online webinars that were free and accessible globally through the Zoom platform. The webinars were promoted online via WPMN’s social media and through word of mouth. Each webinar lasted at least one hour and was led by female surgeons and surgeons from WP backgrounds, focusing on a specific surgical specialty-Orthopaedics, General Surgery, Vascular, ENT and Cardiothoracics. Each webinar was linked to specific learning objectives, which included gaining insight into training pathways and career guidance in the chosen speciality, qualities required to overcome barriers and the importance of WP awareness, an understanding of the variety and complexities of the speciality and the positive impact of surgeries. Case studies and research studies were used to achieve these learning objectives. Every webinar had time dedicated to exploring the speakers’ distinctive WP experiences and resultant barriers to surgical careers and medical education with interactive Q&A.

A diverse range of speakers were recruited to ensure representation of the targeted audience. Speakers were invited via email and selected based on their prestigious leadership roles (e.g. the first black female consultant surgeon in a specialty in the UK) and their commitment to promoting and supporting diversity in medicine. 100% of the speakers were female, with 60% being consultants and 40% senior registrars. 80% of total speakers identified as being from a WP background and those that didn’t were women surgeons in highly male dominated surgical specialties (cardiothoracics).

### Survey

An anonymous voluntary online survey was designed to evaluate two primary hypotheses: first, that an online webinar series could successfully reach a large diverse audience including a significant proportion of attendees from WP backgrounds; and second, that exposure to WP female surgical role models would disproportionately inspire those from WP backgrounds to pursue a career in surgery compared to non-WP attendees. These hypotheses guided the a priori selection of outcomes, including WP status and inspiration to pursue a surgical career.

The online survey was shared with attendees via the webinar chat function and email (see supplementary material). The survey stated submitted responses were for research and publication purposes. The survey was split into multiple sections collecting both numerical data using 7-point Likert scales and free-text responses. Sections included demographics, WP status (from a list of identified backgrounds as above), insightfulness and usefulness of webinar, perceptions of barriers that may exist for women in surgery and whether the webinar inspired them to pursue a career in surgery. Responses on the WP aspect of the webinar included understanding if the WP role model content was a new and/or positive concept and something viewers would seek more of to explore whether lived experience of systemic barriers (e.g. multiple WP backgrounds) influences how WP-focused content is received.

### Analysis of free text responses

Free text responses were collected for two key questions: what barriers attendees perceive there to be to women from WP backgrounds entering and thriving in surgery plus why attendees felt the WP aspect was a good/bad part of the learning experience. Barriers were grouped into 8 domains for analysis of frequency mentioned: male dominated field, lack of role models/representation, sexism, racism, work-life/family balance, financial, intimidating work environment and less opportunities.

### Webinar outreach

Participants were analysed in terms of geographical and demographic distribution, education level (students, doctors) and widening participation status.

### Statistical analysis

Anonymous results from all 5 webinars were collated into excel and analysed using DataTab software. Descriptive statistics were used to review participant characteristics and survey responses. Categorical data was presented as frequencies and percentages to demonstrate trends. The Likert-scale responses were analysed as ordinal data that was found to be non-normally distributed, and consequently median and interquartile ranges (IQR) were reported.

Where further statistical analysis was appropriate, non-parametric methods were utilised. These included the Mann-Whitney U Test for comparisons between independent groups, the Wilcoxon Test for paired data and the Spearman’s Rank Correlation Coefficient to assess correlations. Effect sizes were interpreted alongside p values to aid interpretation of the strength of associations. Statistical significance was defined as *p* < 0.05.

## Results

**Fig. 1:**
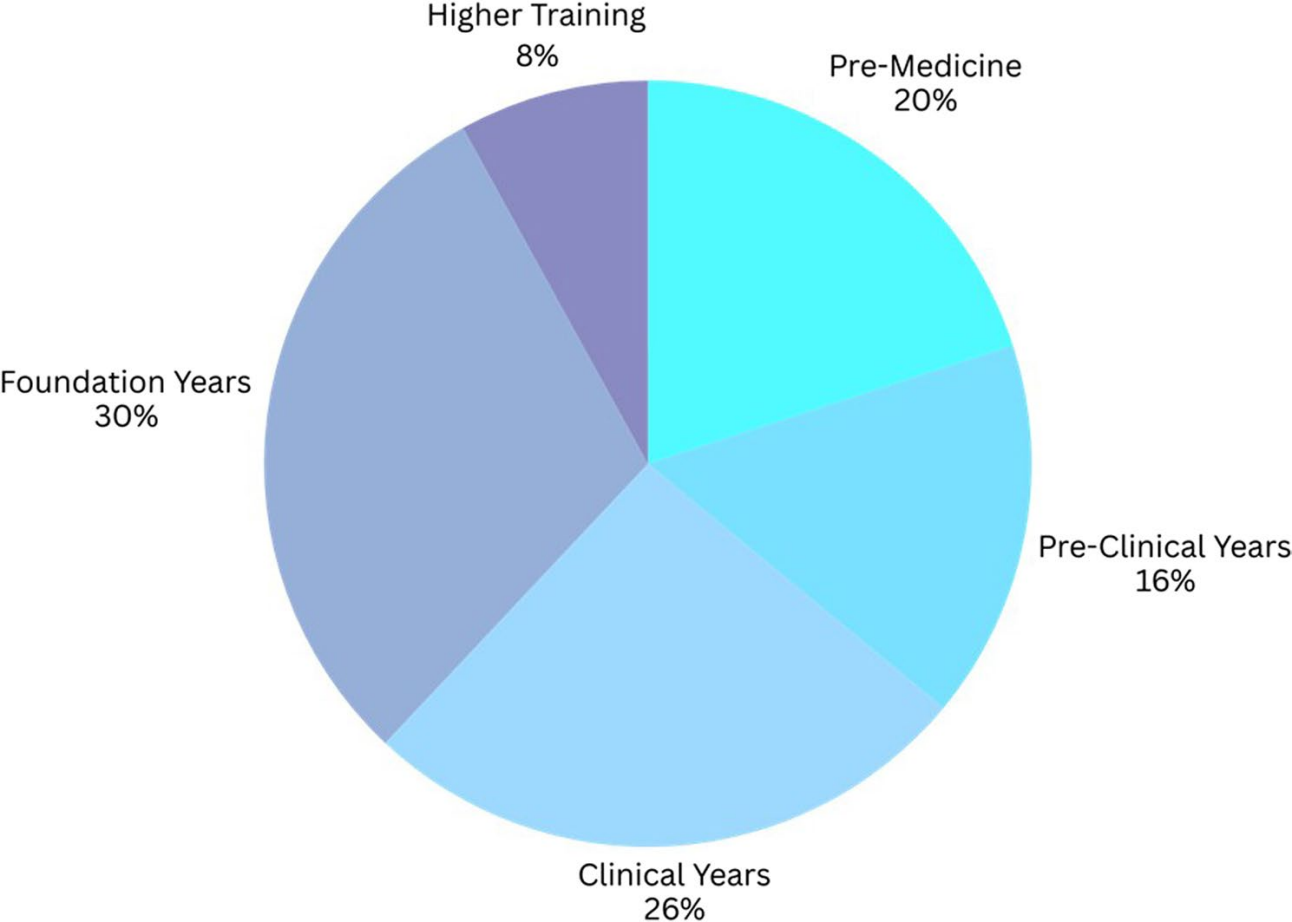
Training level of attendees

Over the course of 5 webinars we had 369 live viewers with an average of 74 viewers per webinar. We received 275 completed surveys amounting to a 75% response rate. As depicted in Fig. 1, the majority of attendees were medical students either in early pre-clinical years or later clinical years (42%) and foundation doctors (30%). The webinars also reached pre-medical students (20%) and higher specialty doctors (8%). The webinar was accessible worldwide with 81% of attendees being UK based while others joined from Europe, Asia, Africa, Australia and North and South America.

**Fig. 2:**
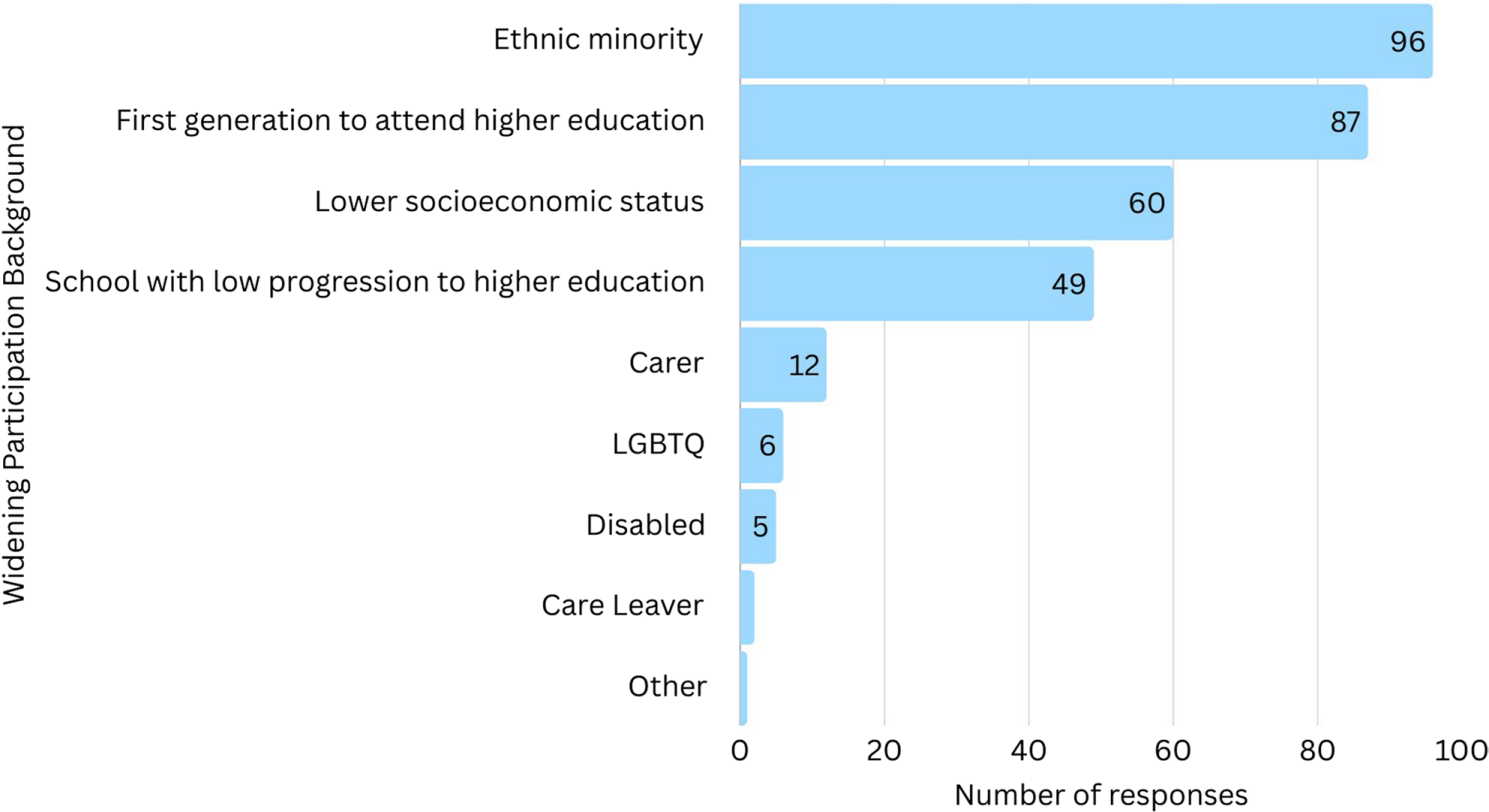
Attendees’ Widening participation (WP) backgrounds

Attendees were asked if they identified as being from a widening participation (WP) background, 60% did, 25% did not and 15% preferred not to say. Figure 2 displays the range of WP backgrounds attendees identified as. Out of the total responses, 35% reported being from an ethnic minority background, 32% were the first generation to attend higher education, 23% identified as being from a low socioeconomic status household and 18% attended a school with low progression to higher education. Among the attendees, 6 identified as members of the LGBTQ+ community, 12 as carers, 2 as care-leavers, and 5 as individuals with a disability. A proportion (27%) of attendees identified as being from more than one WP background with some attendees identifying up to five different WP backgrounds.

When asked the extent to which they agree with the statement “There are many barriers preventing women from widening participation backgrounds entering into a surgical career and thriving as a surgeon” 73% attendees agreed with this statement (Table 1). There was a correlation between the sum of WP backgrounds and the extent to which the attendees agreed with this statement (Spearman’s correlation *r* = 0.22, p = < 0.002).

**Table 1:** Results from the survey showing responses to statements captured on a Likert scale (*n* = 275). Median and interquartile range (IQR) response shown for each statement

*Value given for statistical analysis	Very strongly agree (n)	Strongly agree (n)	Agree (n)	Neutral (n)	Disagree (n)	Strongly disagree (n)	Very strongly disagree (n)	*Median (IQR)
	7	6	5	4	3	2	1	-
‘There are many barriers preventing women from widening participation backgrounds entering into a surgical career and thriving as a surgeon’	36	70	97	34	8	16	14	*5 (5–6)
This talk made me more inspired to pursue a career in surgery	100	73	61	28	2	1	10	*6 (5–7)
	Excellent (n)	Very good (n)	Good (n)	Neither (n)	Poor (n)	Very poor (n)	Extremely poor (n)	*Median (IQR)
*Value given for statistical analysis	7	6	5	4	3	2	1	-
My insight into the experience of Women in Surgery from Widening Participation backgrounds BEFORE the session was/is…	6	20	84	92	52	17	4	*4 (3–5)
My insight into the experience of Women in Surgery from Widening Participation backgrounds AFTER the session was/is…	72	117	75	9	1	1	0	*6 (5–7)
I found the usefulness of this webinar to be…	113	109	49	4	0	0	0	*6 (6–7)
I found the content of this webinar to be…	141	96	34	4	0	0	0	*7 (6–7)
I found the speaker’s presentation style in this webinar to be…	166	84	22	3	0	0	0	*7 (6–7)

When asked to identify barriers in short answer questions, common responses referred to sexism and gender roles, surgery being a male dominated and intimidating work environment, lack of representation and role models, racism, work-life balance and childcare (Table 2).

**Table 2:** Free text survey comments

Question	Response
‘There are many barriers preventing women from widening participation backgrounds entering into a surgical career and thriving as a surgeon’ If you agree with this statement, what are the barriers you perceive there to be?	‘Being a woman in a highly male environment can be intimidating’ ‘Lack of role models’ ‘Fears of racism and sexism, misogyny’ ‘Perception of the field as not being suitable for certain types of people’ ‘Lack of representation’ ‘Poor flexibility in working schedules’ ‘I would perceive that given it has been a male-dominated field for so long, men have had more time to create/develop strong relationships or mentorships that put them ahead as far as opportunities or referrals for positions in surgery go’ ‘Lack of support or misconceptions to those with different cultural backgrounds’ ‘Perception that surgery is less caring/empathetic’ ‘Pressure to enter medical fields where females are more present’ ‘Lack of mentoring, stereotypes, financial barriers and pressures, work-place culture’ ‘So few visible black female surgeons’ ‘Discrimination, microaggressions, the feeling that you might not belong or aren’t good enough’ ‘Derogatory remarks about women who become surgeons and mothers’ ‘Lack of support and empowerment for women entering the medical field’ ‘Preferential treatment of male students/doctors’ ‘Invisible ceiling or prevailing stereotypes that women do not specialise in surgery’ ‘Less access to research opportunities’ ‘Lack of opportunities for women doctors/students to interact with women surgeons’ ‘Sexual behaviours and sexual harassment from senior male surgeons’
Please state why you felt the WP aspect was or was not a good learning experience	‘Relatable to me’ ‘Makes you feel heard, also its a huge factor when applying and it needs to be talked about’ ‘It provided insight as to how to overcome difficulties and shows you can still succeed’ ‘As I am not from a WP background myself, I find it important to learn more about the struggles of people from those backgrounds to help foster a more inclusive and diverse environment in the future.’ ‘I have attended very few webinars that focus on the demographics or barriers of getting into this industry. I think, like this webinar, it’s better to hear from an actual female and/or minority surgeon that has truly experienced this and can give real insight’ ‘It validated my fears as a woman from an ethnic minority group but also provided ways in which I can combat the difficulties I face’ ‘I’m from a Wams background so it helps me see how I can get into surgery even though it may be harder’ ‘It makes it more realistic. Many of the non-WP talks I attend don’t consider the possible barriers an individual might face coming from a WP background’ ‘Helps students to see people like them fulfilling job roles that are from similar backgrounds to us’ ‘Just simply made me feel like I could do it too’

Table 3 shows responses to the motivation behind attending the webinars. The most common answers were the presence of a female surgeon (77%), overall surgical theme (65%), the surgical specialty theme (44%) and the widening participation status of the speaker (38%). Some attendees stated more than one reason for attendance. A third of attendees (33%) had never attended a webinar or conference where the speaker discussed their widening participation background. Of those who had, 37% had attended an event by WPMN previously. Additionally, there was a positive correlation between previous attendance at WPMN events and attendance at events/conferences where the speaker discusses their WP background (Spearman’s correlation p = < 0.001, *r* = 0.27). A Mann–Whitney U test was conducted to compare prior attendance at WP-related events between participants from non–WP backgrounds and those from WP backgrounds. The results indicated a statistically significant difference between the two groups at the 5% significance level (*p* = 0.021), with those from WP backgrounds reporting higher levels of attendance. The effect size was small (*r* = 0.15), suggesting that while the difference between groups was statistically significant, the magnitude of the effect was modest.

**Table 3:** Reasons for attending the webinar; participants were able to choose more than one option (*n* = 275)

Question	Response options					
Why did you attend the webinar?	Female surgeon speaker	Overall surgical theme	Specific surgical speciality	Speaker was from a widening participation background	Certificate of attendance	Quality of portfolio/CV of speaker
Number of responses	213 (77%)	178 (65%)	120 (44%)	104 (38%)	57 (21%)	56 (20%)

Most attendees (95%) found the widening participation aspect of the webinar series to be a positive addition to the learning experience. Table 2 shows examples of comments related to the WP experience. The majority (54%) of those that did not realise there was a WP teaching element were not from widening participation backgrounds. After attending the webinar, 84% of attendees agreed that they were more inspired to pursue a career in surgery. A Mann–Whitney U test was conducted to compare responses to this question between participants from non–WP backgrounds and those from WP backgrounds. The results indicated a statistically significant difference between the two groups at the 5% significance level, with those from WP backgrounds reporting higher levels of inspiration (p = < 0.001). The effect size was of medium magnitude, suggesting that WP background status has a meaningful, though not large, impact on participants’ reported inspiration to pursue a surgical career post webinar.

A statistically significant increase in insight into the experiences of women in surgery from widening participation backgrounds was found, with median Likert responses rising from neither good nor poor (pre-webinar) to very good (post-webinar) (Wilcoxon signed-rank test, *r* = 0.74, *p* < 0.001). Feedback relating to the delivery and reception of the webinars was highly positive, with 81% rating the usefulness of the webinar as excellent or good, 86% rating the content as excellent or good, and 91% rating the speakers’ presentation style as excellent or good.

## Discussion

A need for a more inclusive surgical workforce where women are seen as surgeons and leaders is evident. Despite the benefits for patients treated by female surgeons and diverse teams, representation of women as surgeons remains challenging. This study aimed to assess if the use of an online webinar series would be a plausible way to provide diverse role models to those from diverse backgrounds in the hope of inspiring attendees to pursue a career in surgery.

### Webinar outreach

We were able to design and deliver a wide- reaching international educational series with over 350 live viewers. Its global viewers and high questionnaire response rate highlighted the current interest in WP and women in surgical education. We know that intersectionality plays a huge role in limiting the successes of female trainees and preventing women from attaining leadership and academic positions (here defined as roles held alongside clinical practice, such as University appointments at Senior Lecturer and Professor level, or Institutional Leadership roles such as Departmental Chair or College Board positions) [3, 4]. We successfully met our aims to recruit diverse speakers and reach diverse attendees.

The webinars were attended by many from WP backgrounds. There was also diversity in the education level of attendees from pre-medical students to specialty registrars. This provides evidence that the webinars were successful in reaching the targeted audience. The diversity amongst attendees was further represented in our speakers, which was important to provide role models representative of our targeted audience.

The ease of global access without cost allowed viewers to voluntarily tune into the talks which were delivered by a woman in a position of leadership in a specialty of interest whilst working busy on call rotas. As the webinar series catered for all, with a breadth of seniority of attendees, it championed the thought that role models are important at all stages of a medical career; from giving you that initial inspiration to choose a specialty, to supporting you through applications, navigating work as a surgical trainee amongst family and social commitments and reassuring you that those leadership and research positions are attainable [13–15].

### Existing perceptions and motivation

The majority of attendees agreed with the statement ‘there are many barriers preventing women from widening participation backgrounds entering into a surgical career and thriving as a surgeon’ on a likert scale. This was furthered by a positive correlation between the number of WP characteristics of an attendee and the likelihood of agreeing to the statement, suggesting barriers are perceived more readily in those from more diverse backgrounds. Arguably due to lived experiences and the impact of intersectionality, as evidenced in the literature when comparing perceived career barriers between men and women of different ethnicities [16]. Free-text responses commonly identified systemic barriers such as sexism, racism, gender expectations, work-life balance, and lack of representation and role models as a deterrent to pursuing a career in surgery. These barriers are well documented and reflect current literature that have sought to identify structural and cultural inequities in surgical training pathways [17, 18].

Attendees’ motivation to attend the talks were primarily led by female surgeon speakers secondly by surgical theme and thirdly due to the widening participation status of the speaker. Together, these responses propose the lack of- and desire for - diverse role models and female representation. At present medical students and early years doctors often rely on the diversity of the university and hospital teams they work in to provide role models. Women now outnumber men with regards to the number of doctors working in the UK but this ratio dramatically reverses when looking at the surgical cohort [1, 19]. This decreases dramatically at consultant level [9, 20] making it sometimes rare to work alongside women surgical consultants in specialties of interest. Evidence suggests female students and doctors are more likely to follow in the footsteps of other women by selecting a career such as surgery, if exposed to more female residents or faculty i.e. having female role models [21, 22]. If our aim is to increase the number of women in surgery and increase the number of women from WP backgrounds in surgery, it follows that providing diverse women role models through webinars is one method of doing so.

### Impact

Surveys showed the webinars significantly increased insight into the experiences of WP women in surgery and were highly rated by attendees. The majority of attendees (irrelevant of being a WP or not) reported high levels of inspiration to pursue a career in surgery post webinar, conveying the effectiveness of the pedagogical principles of WP related content that benefited all learners and was not exclusive to underrepresented groups. Those from WP backgrounds were statistically more inspired, which further demonstrates the importance of representation when the goal is to inspire those from diverse backgrounds. Based on their systematic review findings, Gladstone et al. [23] formed four recommendations to maximise the effectiveness of STEM role models - one being prioritising role models from underrepresented groups. Interestingly they found role models from underrepresented groups to have the broadest positive effect on all students regardless of their WP status. They further suggested the use of videos to avoid burdening such role models whilst being as effective as live interactions.

The void in representation within surgical medical education was illustrated by a third of attendees having never experienced educational events that discussed WP background. Participants from WP backgrounds were significantly more likely to have attended more WP-focused events compared with those without such backgrounds. Attendance patterns indicate a strong association between prior participation in WPMN events and engagement with other widening participation (WP) events. This suggests a sustained demand for WP-oriented content, particularly among individuals already embedded within WP networks, highlighting both the relevance and the potential reinforcement of WP engagement within this community. Most attendees found the WP element of the series to be positive. The majority of those who did not acknowledge the WP aspect identified as not being from a WP background, which suggests the presence of a lack of awareness amongst those from non-WP backgrounds. Additionally, expanding outreach to increase exposure of non-WP individuals to role models from underrepresented groups is warranted and is important given previously referenced evidence that such representation has been shown to have the broadest positive effects on learners, not limited to those from WP backgrounds [23].

When asked ‘what was done well’ attendees responses included clear structure, engaging delivery, and well-designed case presentations. Speakers were described as inspiring and relatable, providing honest insights into surgical careers and representation in the field. Participants highlighted the value of the interactive Q&A sessions, practical career advice, and the balanced focus on clinical knowledge and personal experience. Overall, the sessions were viewed as informative, well organised, and motivating.

The survey also identified possible areas for improvement when planning and executing further webinar series aimed at WP students. With regards to the clinically focussed aspect of the webinar participants suggested incorporating more surgical case studies, patient examples, and visual material to enhance clinical understanding. In relation to career advice and the WP aspect, attendees would like more practical career guidance such as advice on portfolio building, applications, and interviews, tailored advice for international medical graduates and medical students, and discussion of work–life balance and personal challenges. Many liked the Q&A aspect and requested more time for this to allow fuller discussion and interaction.

### Limitations

A limitation of the study was the absence of follow up to evaluate the long-term impact. Selection bias should also be acknowledged, as individuals who chose to register for the webinars may have already held a pre-existing views and interest in surgery or diversity initiatives, meaning the audience may not be representative of the broader trainee population. Similarly, response bias among those who chose to complete the survey may have skewed results in a positive direction, as those with more favourable impressions of the webinars may have been more motivated to respond.

### Future research

Our next challenge will be to determine whether the initial positive impact of the webinars as evidenced by the survey led to long-term successful pursuit of a career in surgery. That said, the immediate feedback from this study has been promising. Women in surgery and WP related initiatives need to focus on sustainability, assessing the success of interventions in attracting diverse doctors into surgery, supporting them to be successful trainees and promoting them to positions of leadership.

Future iterations of this work could explore the integration of similar educational series into both undergraduate and postgraduate training, with a particular focus on early year medical students — the stage at which initial specialty inspiration is commonly formed. Incorporating digital platforms such as webinars and access to role models into the medical curriculum at this early stage, raising WP awareness and providing examples of successful career pathways, may further accelerate positive change towards a more inclusive and diverse surgical workforce. Longitudinal mentorship models developed from initiatives such as this could then provide sustained support for WP trainees as they progress, with a particular focus on areas of greatest inequity such as academia and leadership.

This approach would also complement existing initiatives such as the Royal College of Surgeons WinS Network [10], which is a national organization working to promote surgery as a career for women, and support professional career progression for women who have already chosen a career in surgery. The network provides a mentorship platform pairing mentees and mentors across varying grades. The current WinS model is primarily accessible to those already within postgraduate training, excluding medical students at present. The webinar series described in this study is therefore well positioned to extend this support earlier, serving both as a means for students to identify potential future mentors whose experiences resonate with their own, and as an accessible on-demand resource of career guidance and lived experience.

## Conclusion

This study demonstrated the success of a WP charity in creating an accessible, insightful and useful educational tool that increases the visibility of female surgical role models. The study reached those from WP backgrounds across their medical career and highlighted a demand for more WP related educational content. The study’s method of providing visibility of women in surgery would arguably work in harmony with other current initiatives such as The WinS network [10]. However, more research is required to assess the long-term sustainability and success of women in surgery initiatives in creating meaningful changes in career outcomes.

## Supplementary Information

Supplementary Material 1

## Data Availability

All data is included in the manuscript in the form of tables and figures. The online survey is included in the supplementary material.

## Abbreviations

ENT: Ear, Nose and Throat
Non-WP: Non widening participation
Q&A: Question and answer
WP: Widening participation
WPMN: Widening Participation Medics Network
WinS: Women in Surgery
