# Supplementary material for: The power of webinars: expanding diverse female representation and role models in surgery
Source: BMC Med Educ. 2026 May 1;26:993. doi: 10.1186/s12909-026-09326-0 (PMC13277104; doi:10.1186/s12909-026-09326-0)
Supplement: Supplementary file 1 — Supplementary Material 1 [file 12909_2026_9326_MOESM1_ESM.pdf]

Thank you for attending Widening Participation Women in Surgery...

Please give us your honest feedback on this talk as it will be used to improve the lecture series and as evidence of teaching for the presenter!

By filling in this feedback form, you are agreeing to your anonymised data to be collected and used by WPMN for research and publication purposes and to be shared with partnering organisations to enhance widening participation education.

Names and email addresses are only used to provide certificates.
The Royal College of Surgeons of England has awarded up to 1 CPD points for this event.
This will be stated on your certificate.

* Indicates required question

1. Email *

____

Demographics

2. What stage of medical education are you? *

Mark only one oval.

- Sixth form/college
- School graduate applying to medicine
- University graduate applying to medicine
- Mature student/ Career change applying to medicine
- Foundation Year
- Year 1 Medical Student
- Year 2 Medical Student / Graduate Entry Year 1
- Year 3 Medical Student / Graduate Entry Year 2
- Year 4 Medical Student / Graduate Entry Year 3
- Year 5 Medical Student / Graduate Entry Year 4
- Intercalation year
- FY1
- FY2
- Higher training
- Other: ______________________

3. Which university do you attend? *

Mark only one oval.

- I am a DOCTOR
- I have NOT yet attended a medical school/university
- University OUTSIDE of the UK
- Aberdeen University
- Anglia Ruskin University
- Aston University
- Barts University
- Bangor University
- Birmingham University
- Brighton and Sussex University
- Bristol University
- Brunel University
- Buckingham University
- Cambridge University
- Cardiff University
- Central Lancashire University
- Dundee University
- Edge Hill University
- Edinburgh University
- Exeter University
- Glasgow
- Hull York University
- Imperial College London University
- Keele University
- Kent and Medway Medical School
- King's College London
- Lancaster University
- Leeds University
- Leicester University
- Liverpool University
- London School of Hygiene & Tropical Medicine
- Manchester University

- Newcastle University
- Norwich Medical School
- Nottingham University
- Oxford University
- Plymouth University Peninsula Schools of Medicine and Dentistry
- Queen's University Belfast
- Sheffield University
- Southampton University
- St Andrews University
- St George's University
- Sunderland University
- Swansea University
- University College London
- Warwick University
- Ulster University
- Other: ______________________

4. Which deanery or area do you study/work in? *

Mark only one oval.

- East Anglia
- Essex, Bedfordshire & Hertfordshire (EBH)
- Leicester, Northamptonshire & Rutland (LNR)
- London & KSS: (North Central and East London; North West London; South Thames)
- North West of England
- Ireland
- Northern
- Oxford
- Peninsula
- Scotland
- Severn
- Trent
- Wales
- Wessex
- West Midlands (Central, North & South)
- Yorkshire & Humber
- Europe
- North America
- South America
- Africa
- Asia
- Australia
- Other: __________

Feedback

5. My insight into the experience of Women in Surgery from Widening Participation backgrounds was/is... *

Mark only one oval per row.

	Extremely poor	Very poor	Poor	Neither good nor poor	Good	Very good	Excellent
**Before webinar**	⬭	⬭	⬭	⬭	⬭	⬭	⬭
**After webinar**	⬭	⬭	⬭	⬭	⬭	⬭	⬭

6. I found the usefulness of this webinar to be... *

Mark only one oval per row.

	Extremely poor	Very poor	Poor	Neither good nor poor	Good	Very good	Excellent
.	⬭	⬭	⬭	⬭	⬭	⬭	⬭

7. I found the content of this webinar to be... *

Mark only one oval per row.

	Extremely poor	Very poor	Poor	Neither good nor poor	Good	Very good	Excellent
.	⬭	⬭	⬭	⬭	⬭	⬭	⬭

8. I found the speaker's presentation style in this webinar to be... *

Mark only one oval per row.

	Extremely poor	Very poor	Poor	Neither good nor poor	Good	Very good	Excellent
.							

9. To what degree to you agree with the following statement "This talk made me more inspired to pursue a career in surgery" *

Mark only one oval.

- Very strongly disagree
- Strongly disagree
- Disagree
- Neutral
- Agree
- Strongly agree
- Very strongly agree

10. What was done well in the webinar? *

11. Please suggest at least one improvement *

12. What educational/career topics would you like to see in our future webinars?

Widening Participation and Diversity in Surgery

13. Which widening participation background are you from? *

Check all that apply.

- [ ] I am not from a widening participation background
- [ ] Prefer not to disclose
- [ ] Attend/ed a school with low progression into higher education
- [ ] Are/were the first generation of my family to consider higher education
- [ ] From an underrepresented ethnic group
- [ ] From a low socio-economic group
- [ ] Care leaver
- [ ] Carer
- [ ] Disabled
- [ ] Other: ______________

14. To what extent do you agree with this statement - 'There are many barriers preventing women from widening participation backgrounds entering into a surgical career and thriving as a surgeon' *

Mark only one oval.

- Very strongly disagree
- Strongly disagree
- Disagree
- Neutral
- Agree
- Strongly agree
- Very strongly agree

15. If you agree, what are the barriers you perceive there to be?

16. How many webinars/conferences have you attended where the speaker/s discuss their widening participation background? (excluding WPMN events) *

Mark only one oval.

- 0
- 1-3
- 4-6
- >7

17. Did you find the widening participation (WP) aspect of the webinar a good addition to the learning experience? *

Mark only one oval.

- Yes - it was a positive addition to the surgical theme
- What widening participation aspect?
- No - it did not add to the learning experience
- No - it was a negative addition to the learning experience

18. Please state why you felt the WP aspect was or was not a good learning experience?

WPMN Events

19. Have you attended a WPMN event before? *

Mark only one oval.

- Yes
- No

20. Why did you choose to attend this webinar? *

Check all that apply.

- [ ] Overall surgical theme
- [ ] Specific surgical speciality theme
- [ ] Female surgeon speaker
- [ ] Speaker was from a widening participation background
- [ ] Quality of portfolio/CV of the speaker
- [ ] Heard/experienced previous webinars that were worthwhile watching
- [ ] Certificate
- [ ] Other: ______

This content is neither created nor endorsed by Google.

Google Forms
